# Enhanced host–guest interaction between [10]cycloparaphenylene ([10]CPP) and [5]CPP by cationic charges

**DOI:** 10.3762/bjoc.20.38

**Published:** 2024-02-23

**Authors:** Eiichi Kayahara, Yoshiyuki Mizuhata, Shigeru Yamago

**Affiliations:** 1 Institute for Chemical Research, Kyoto University, Uji 611-0011, Japan

**Keywords:** charge-transfer, cycloparaphenylene, dication, host–guest chemistry

## Abstract

A dication of [5]cycloparaphenylene ([5]CPP^2+^) was selectively encapsulated by neutral [10]CPP to form the shortest double-layer carbon nanotube, [10]CPP⊃[5]CPP^2+^. While the same host–guest complex consisted of neutral CPPs, [10]CPP⊃[5]CPP, was already reported, the cationic complex showed an about 20 times higher association constant in (CDCl_2_)_2_ at 25 °C (10^3^ mol L^−1^). Electrochemical and photophysical analyses and theoretical calculations suggested the partial electron transfer from [10]CPP to [5]CPP^2+^ in the complex, and this charge-transfer (CT) interaction is most likely the origin of the higher association constant of the dicationic complex than the neutral one.

## Introduction

**Figure 1:**
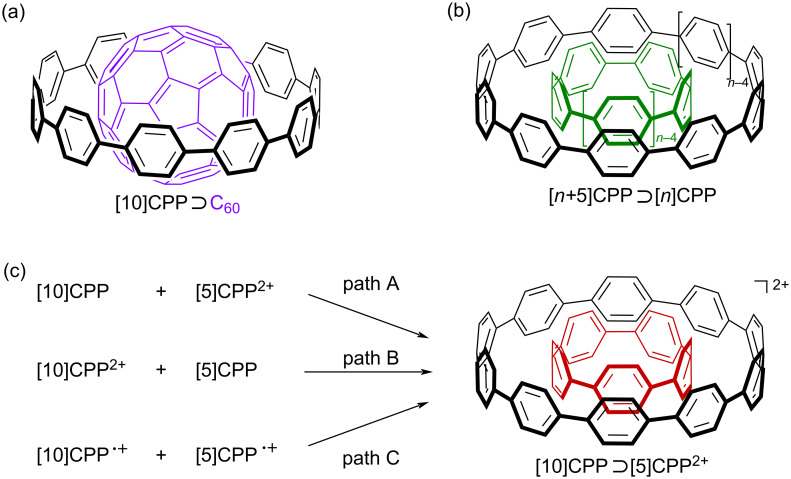
Structures of a) [10]CPP⊃C_60_, (b) [*n+5*]CPP⊃[*n*]CPP, and (c) [10]CPP⊃[5]CPP^2+^ (this work).

Since the first bottom-up organic synthesis of cycloparaphenylenes (CPPs) [1–7], which are the carbon nanorings with the shortest possible structural constituent of armchair carbon nanotubes (CNTs), a new science of cyclic nanocarbons has emerged through synthesizing new CPP analogs [4–8] and unveiling their unique physical properties, such as size-dependent photophysical [9–15] and redox properties [16–21]. The other, and one of the most exciting, functions of CPPs derived from the ring structure is their host function. After our first report on the selective encapsulation of C_60_ by [10]CPP (the number in the brackets is the number of paraphenylene units in the CPP) (Figure 1a) [22], the concave inner surface of CPPs was found to interact with a variety of molecules with convex surfaces through π–π interactions [23–26] and CH–π interactions [27–30], forming the corresponding host–guest complexes. In addition, the concave outer surface of the CPP also interacts with the convex surface of the CPP (Figure 1b) [31–33], resulting in the formation of the shortest possible double-layer CNTs. Furthermore, Isobe and co-workers also showed that the carbon nanorings with the simplest structural unit of chiral CNTs, such as cyclochrysenylene [34], cyclonaphthylenes [35], and cycloanthanthrenylene [36], are also excellent hosts of fullerenes with exceptionally high binding constants. These results open up new possibilities for the fabrication of supramolecular structures based on the non-covalent interactions using carbon nanorings [37–38].

Despite the unique structure of the host–guest complexes, however, their electronic structures are not very attractive. This is because the complex formation is driven by van der Waals interactions, and no new electronic states are created by electronic perturbations between the host and the guest, except for a few examples using special fullerenes as guest molecules, i.e. [11]CPP⊃La@C_82_ [39], and [10]CPP⊃Li^+^@C_60_ [40], and [10]CPP⊃(C_59_N)_2_ [41], or applying high pressure (6 GPa) in the complexation between [9]CPP and C_60_ [42]. In these examples, partial charge transfer (CT) from the CPP host to the guest was observed, but the degree of CT was limited. Furthermore, no clear effects of CT on the physical properties have been reported.

We have already reported that two-electron oxidation of [*n*]CPPs yields dications, [*n*]CPPs^2+^ [17,21,43], which are unusually stable due to the presence of in-plane aromaticity derived from the ring structure [19]. Therefore, we speculated that the CPP dication could be used as a host or a guest to alter the electronic state of the corresponding host–guest complex. Here, we report on the size complementary formation of the host–guest complex between [10]CPP and the dication of [5]CPP, [10]CPP⊃[5]CPP^2+^ (Figure 1c). The association constant, *K*_a_, determined by ^1^H NMR titration, was about 20 times higher than that of the host–guest complex consisting of neutral [10]CPP and [5]CPP, [10]CPP⊃[5]CPP [31]. While the ^1^H NMR analysis also supported the closed-shell electronic structure of both the host and the guest, neutral [10]CPP and [5]CPP^2+^, respectively, a partial CT from the host to the guest was suggested by the photophysical and electrochemical analyses and DFT calculations. Therefore, this CT is most likely the origin of the increased *K*_a_ value. We also discuss the charged double-layer structure, as determined by X-ray crystallographic analysis.

## Results and Discussion

**Figure 2:**
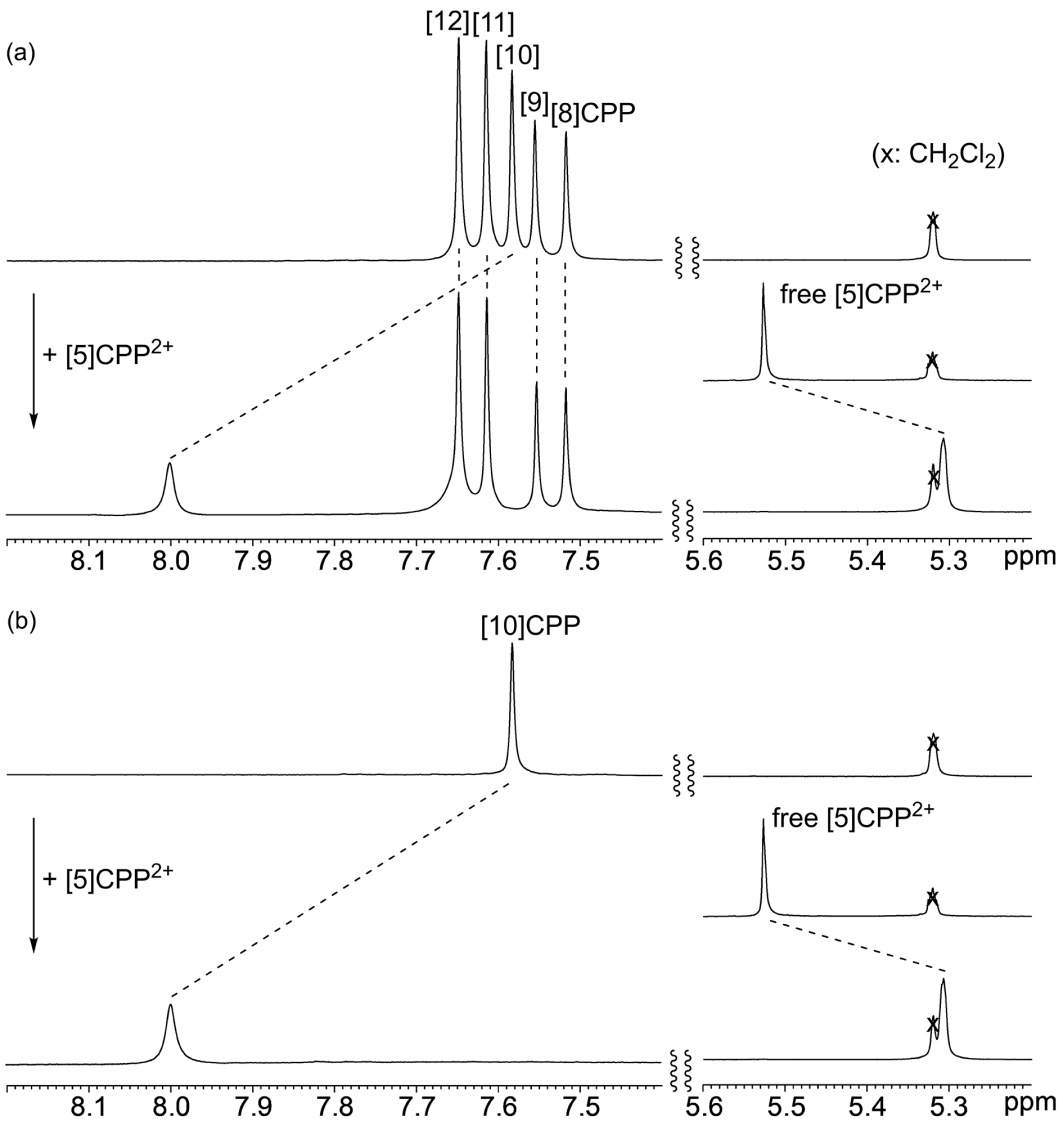
^1^H NMR spectra (CD_2_Cl_2_, 25 °C) of a) a mixture of [8]–[12]CPPs and [5]CPP^2+^[B(C_6_F_5_)_4_^−^]_2_ before and after the addition of [5]CPP^2+^[B(C_6_F_5_)_4_^−^]_2_, and b) [10]CPP and [5]CPP^2+^[B(C_6_F_5_)_4_^–^]_2_ before and after the addition of [5]CPP^2+^[B(C_6_F_5_)_4_^−^]_2._

The size-complementary interaction between CPP^2+^ and neutral CPP was first examined by adding a solution of [5]CPP^2+^[B(C_6_F_5_)_4_^−^]_2_ in CD_2_Cl_2_ to a mixture of [8]-, [9]-, [10]-, [11]-, and [12]CPPs in CD_2_Cl_2_ at 25 °C (Figure 2a). In the ^1^H NMR spectrum of the resulting mixture, only the signals of [10]CPP and [5]CPP^2+^ shifted downfield by about 0.44 ppm and upfield by about 0.23 ppm, respectively, with peak broadening. The other spectra derived from [8]-, [9]-, [11]-, and [12]CPPs did not change at all. A separate experiment mixing an equimolar amount of [10]CPP and [5]CPP^2+^[B(C_6_F_5_)_4_^−^]_2_ gave the same result (Figure 2b), indicating a size-complementary interaction between [5]CPP^2+^ and [10]CPP. The same size selectivity was observed for the complex formation between neutral CPPs [31]. As the protons in the ^1^H NMR of [10]CPP^2+^ and neutral [5]CPP resonate at 4.72 and 7.84 ppm, respectively [21], the observed chemical shifts of the complex indicate that the complex formation occurs between neutral [10]CPP and [5]CPP^2+^ forming [10]CPP⊃[5]CPP^2+^ (Figure 1c, path A) [21]. The absence of signals in the ESR measurements at room temperature also suggests the formation of complexes with a closed-shell electronic structure.

The same complex was formed regardless of the oxidation state of the starting CPPs. Thus, when neutral [5]CPP was mixed with [10]CPP^2+^ (SbCl_6_^−^)_2_, only the signals at 8.0 and 5.3 ppm corresponding to [10]CPP⊃[5]CPP^2+^ were observed (Figure 1c, path B), suggesting a quick electron transfer from [5]CPP to [10]CPP^2+^ has occurred. The same complex was also formed by mixing a 1:1 mixture of radical cations of [5]- and [10]CPP, [5]CPP^•+^ (SbCl_6_^−^) and [10]CPP^•+^ (SbCl_6_^−^), respectively (Figure 1c, path C). The observed results can be explained by two reasons; one is the oxidation potentials of [10]- and [5]CPPs. In sharp contrast to linear π-conjugated molecules, CPPs with shorter conjugations have lower oxidation potentials than larger CPPs, and the first and the second oxidation potentials of [5]CPP are by 0.69 and 0.51 V lower than those of [10]CPP, respectively [43]. The other is the stability of the CPP dications. Since the dications are stabilized by in-plane aromaticity, the single-electron transfer from [10]CPP to [5]CPP^2+^ to form [10]CPP^•+^⊃[5]CPP^•+^ is energetically unfavorable.

The association constant (*K*_a_) between [10]CPP⊃[5]CPP^2+^ [B(C_6_F_5_)_4_^−^]_2_ in 1,1,2,2-tetrachloroethane-*d*_2_ (TCE-*d*_2_) at 50 °C was determined to be 1.07 × 10^3^ L·mol^−1^ by ^1^H NMR titration experiments (see Supporting Information File 1, Figure S1), indicating that the complexation was exergonic with a Δ*G* = −19 kJ mol^−1^. This value is about 20 times higher than that of the neutral complex, [10]CPP⊃[5]CPP (*K*_a_ = 0.053 × 10^3^ L·mol^−1^ in TCE-*d*_2_ at 50 °C, Δ*G* = −11 kJ mol^−1^) [31]. The increased *K*_a_ is most likely attributed to the ionic interaction caused by the presence of the cationic species, as will be discussed below. To clarify the effect of the polar interaction, the host–guest complexation was studied in a polar solvent, such as nitrobenzene and acetonitrile. However, the low solubility and stability of the dication in polar solvents prevented confirmation of the solvent effect.

**Figure 3:**
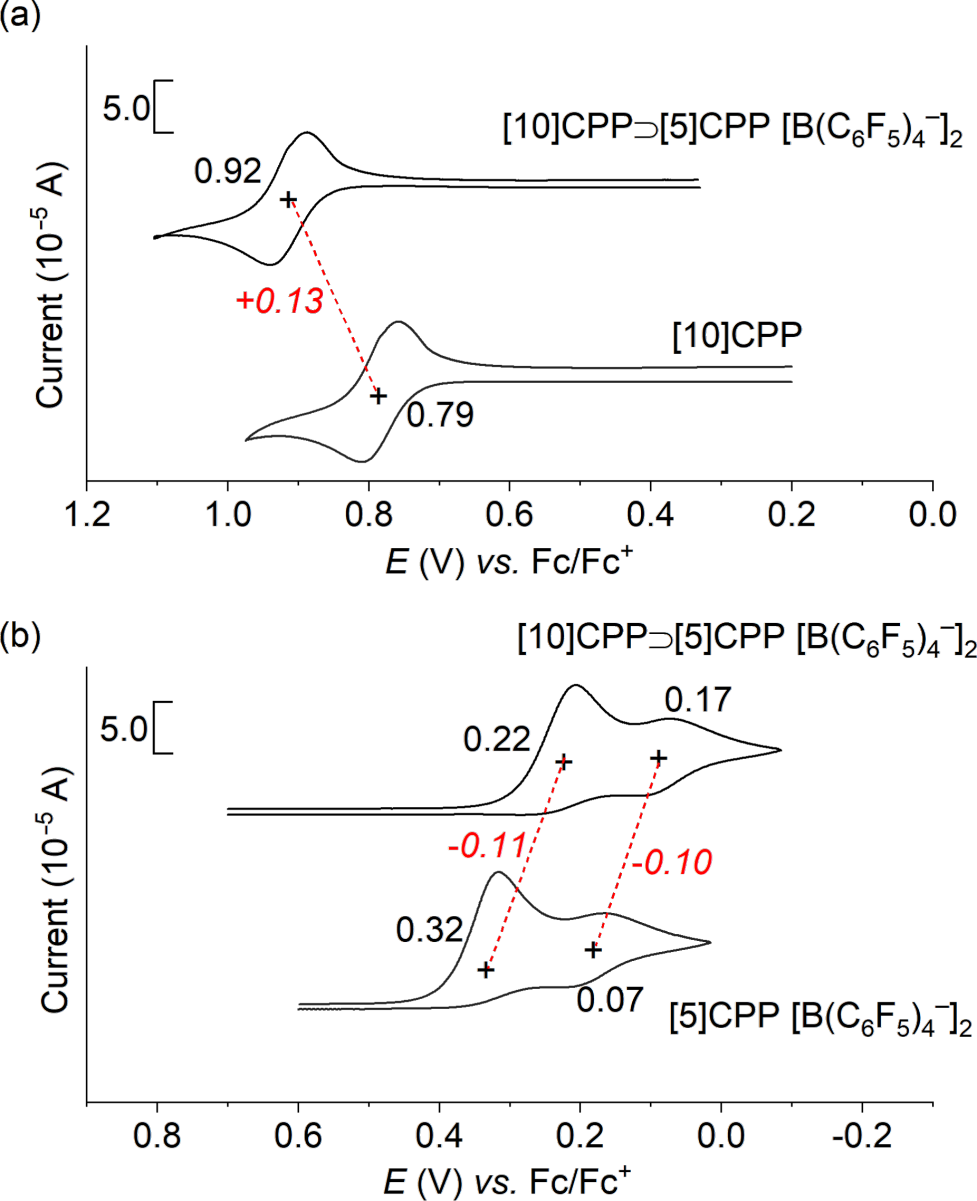
Cyclic voltammograms of [10]CPP⊃[5]CPP^2+^[B(C_6_F_5_)_4_^−^]_2_, [10]CPP, and [5]CPP^2+^[B(C_6_F_5_)_4_^−^]_2_ in Bu_4_N^+^ B(C_6_F_5_)_4_^−^/1,2-dichloroethane upon scanning in (a) positive and (b) negative directions.

To clarify the additional interaction in the complex, the electrochemical analysis of the [10]CPP⊃[5]CPP^2+^[B(C_6_F_5_)_4_^–^]_2_ was examined by using cyclic voltammetry, which was performed in a 1,2-dichloroethane solution of a sample containing 0.10 mol L^−1^ Bu_4_N^+^ B(C_6_F_5_)_4_^−^. The cyclic voltammogram of the complex showed one reversible oxidation wave of [10]CPP at 0.92 V versus (vs) ferrocene/ferrocenium couple (Fc/Fc^+^) (Figure 3a), which was positively shifted by 0.13 V from that of neutral, free [10]CPP. Upon scanning into the negative direction, two pseudo reversible reduction waves of [5]CPP^2+^ were observed at 0.22 and 0.17 V vs Fc/Fc^+^ (Figure 3b), which were negatively shifted by 0.11 and 0.10 V from that of free [5]CPP^2+^[B(C_6_F_5_)_4_^−^]_2_. These results suggest the electron density of [10]CPP and [5]CPP^2+^ decreased and increased, respectively, upon complex formation and clearly indicate the partial electron transfer from [10]CPP to [5]CPP^2+^ in the complex.

**Figure 4:**
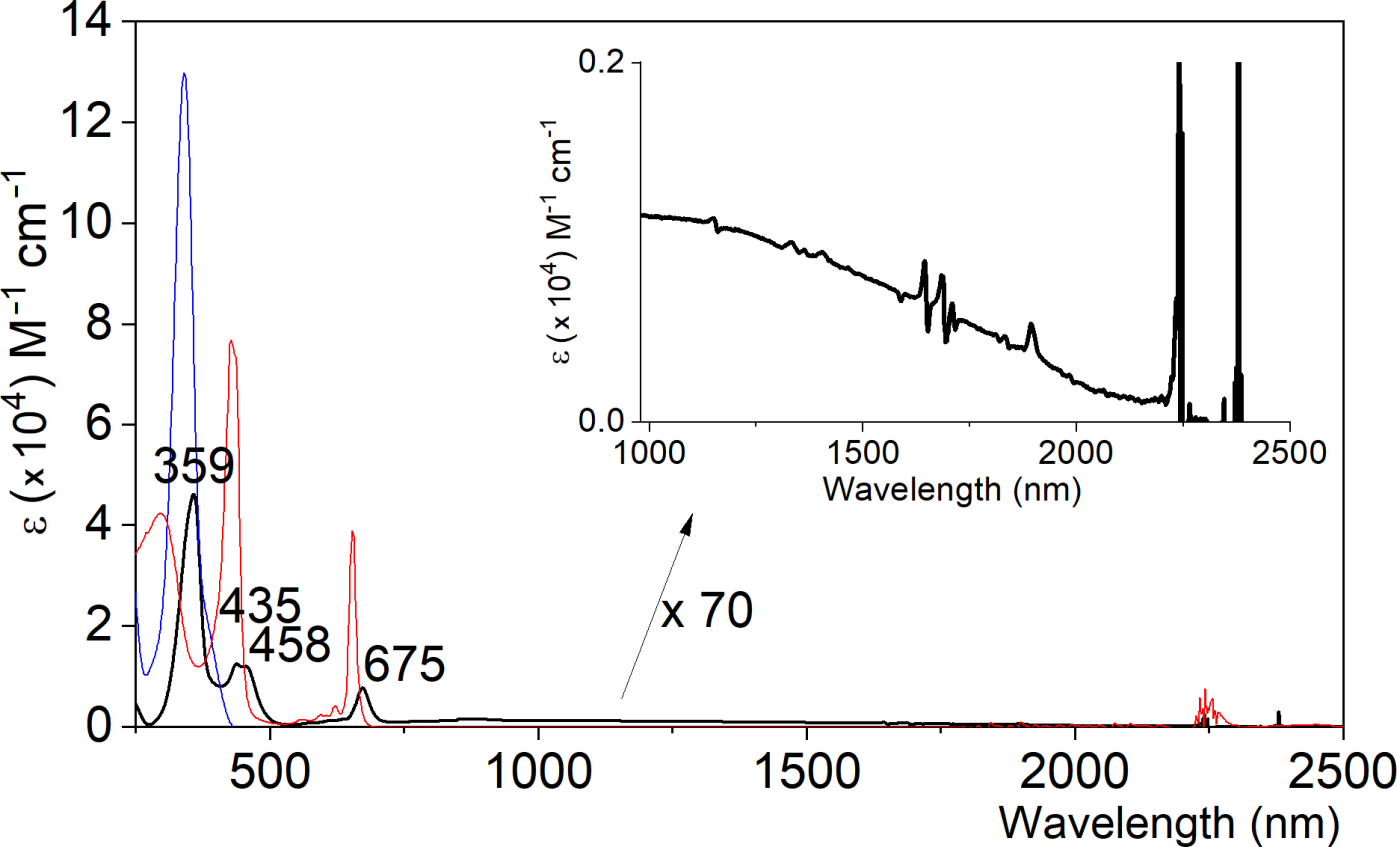
UV–vis–NIR spectra of [10]CPP⊃[5]CPP^2+^[B(C_6_F_5_)_4_^−^]_2_ (black), [10]CPP (blue), and [5]CPP^2+^[B(C_6_F_5_)_4_^−^]_2_ (red) in CH_2_Cl_2_.

The involvement of a CT in the complex was further supported by the UV–vis–NIR absorption spectra. The complex showed several absorption bands in the visible region and characteristic broad absorption bands extending to the NIR region (≈2000 nm) (Figure 4, black line). According to time-dependent density functional theory (DFT) calculations at the ωB97-XD/6-31G* level of theory, the NIR absorption of the most thermodynamically stable complex (see below) can be ascribed to the transition from the HOMO (highest occupied molecular orbital) to the LUMO (lowest unoccupied molecular orbital) with oscillator strengths (*f*) = 0.0001. As the HOMO and LUMO are selectively localized to [10]CPP and [5]CPP^2+^, respectively (see below), this transition corresponds to the CT band.

**Figure 5:**
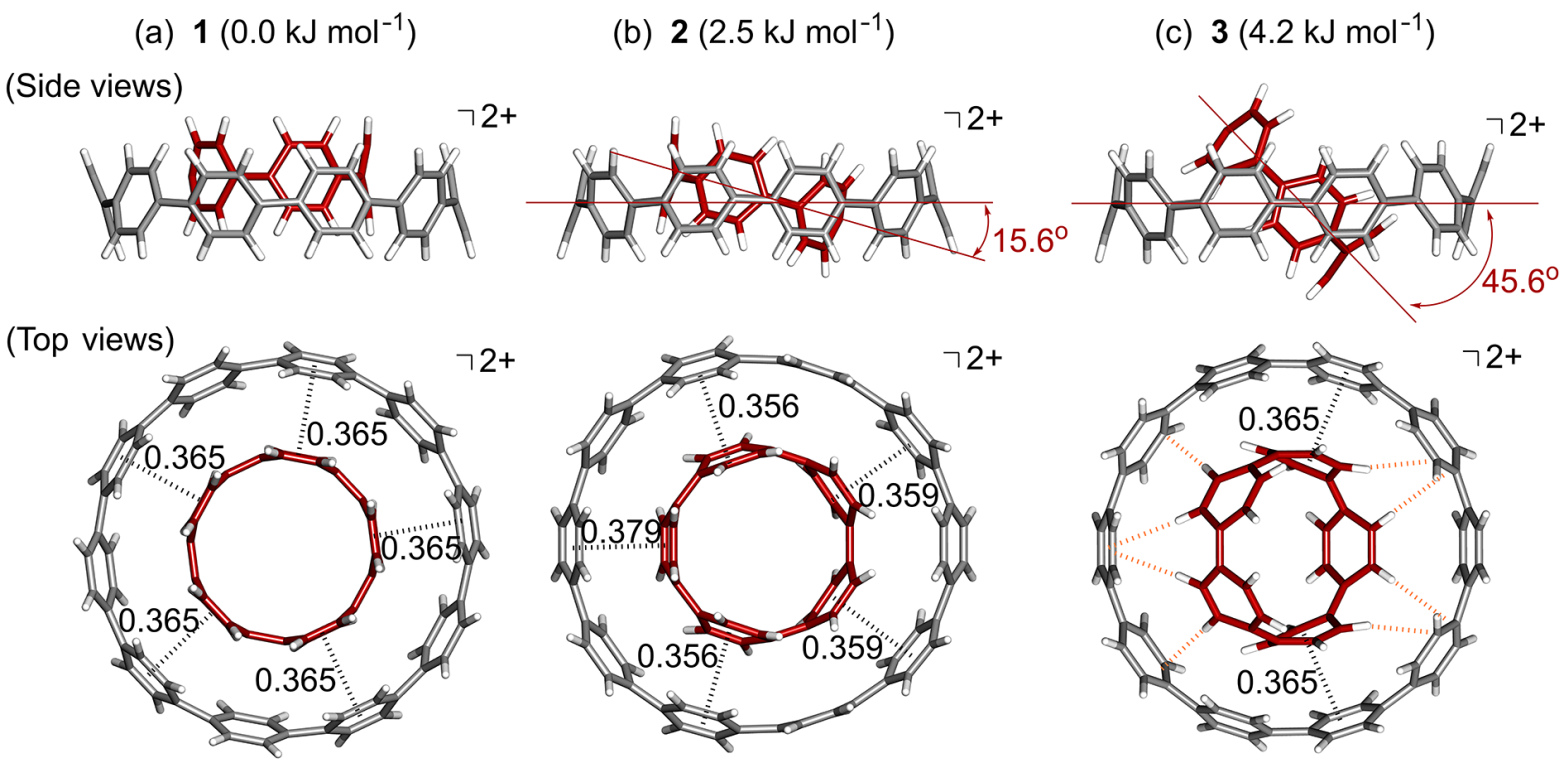
Top and side view of [10]CPP⊃[5]CPP^2+^ complexes a) **1**, b) **2**, and c) **3** obtained by DFT calculation at the ωB97-XD/6-31G(d) level of theory. The distances (nm) between the nearest centroids of a paraphenylene unit of [10]CPP and [5]CPP are shown by the black dotted lines in the top view. The orange dotted lines in the top view of c) show the CH–π interactions between the hydrogen of [5]CPP and the nearest sp^2^ carbon of [10]CPP within 0.30 nm.

The structure and stability of [10]CPP⊃[5]CPP^2+^ were estimated by DFT calculations at the ωB97-XD/6-31G* level of theory. Three isomeric structures were optimized, of which complex **1**, with two CPPs aligned in parallel, is the most thermodynamically stable (Figure 5a). The other two isomers, **2** and **3** (Figure 5b,c), with two CPPs tilted at 15.6° and 45.5°, are 2.5 and 4.2 kJ mol^−1^ less stable than complex **1**, respectively. The stability among the isomers is low, and the activation energy for isomerization should be very low. Therefore, all isomers are expected to be present in solution when the solvent effect is negligible.

Formation of complex **1** in the gas phase was highly exothermic with Δ*G* = −222 kJ mol^−1^ at 298.15 K and 1.00 atm, and this value is about two times higher than that of the complex formation of neutral [10]CPP⊃[5]CPP (Δ*G* = −114 kJ mol^−1^). Therefore, the same trend of the relative stability between [10]CPP⊃[5]CPP^2+^ and [10]CPP⊃[5]CPP was also observed in the gas phase. The significant increase in relative stability in the gas phase compared to the solution state is likely due to the overestimation of electronic interactions due to CT in the absence of a solvent.

Complex **1** possesses a 5-fold rotational axis through the focal point of the complex, and each paraphenylene unit of [5]CPP^2+^ interacts with that of [10]CPP every other unit (Figure 5a). The interfacial distance between the nearest neighbor centroid of a paraphenylene unit of [5]CPP^2+^ and [10]CPP is 0.365 nm, which is very close to the sum of the van der Waals radii of an sp^2^ carbon (0.340 nm) [44]. This mode of interaction is similar to that observed in the complex formed from [10]CPP and C_70_ [24]. In contrast, complex **2** has a less ordered interaction compared to **1**; while each paraphenylene unit of [5]CPP^2+^ interacts with that of [10]CPP every other unit like in **1**, the interfacial distance between the nearest neighbor centroid of a paraphenylene unit is in the range of 0.356–0.379 nm due to the inclination (Figure 5b). Despite the slight structural differences between complexes **1** and **2**, the observed interlayer distances indicate the importance of the π–π interactions in complex formation. In contrast, complex **3** has fewer π–π interactions between [10]CPP and [5]CPP^2+^ than complexes **1** and **2** have. However, several CH–π interactions are observed, as indicated by an orange dotted line (Figure 5c), significantly stabilizing the complex formation. These results confirm the importance of van der Waals interactions for the formation of [10]CPP⊃[5]CPP^2+^.

Mulliken population analysis indicates 10 to ≈15% electron transfer from the host [10]CPP to the guest [5]CPP^2+^ in complexes **1**, **2**, and **3**. While the extent of the CT in the current complexes is slightly greater than that in [11]CPP⊃La@C_82_ (7%) [39], the results are still far from being sufficient for the formation of a radical ion complex, [10]CPP^•+^⊃[5]CPP^•+^. Indeed, the structural properties, as seen from the bond length alternation, showed that the [10]CPP host and [5]CPP guest of the complex were in good agreement with the neutral [10]CPP and [5]CPP^2+^ (see Supporting Information File 1, Table S3). All these theoretical results agree well with the experimental results.

**Figure 6:**
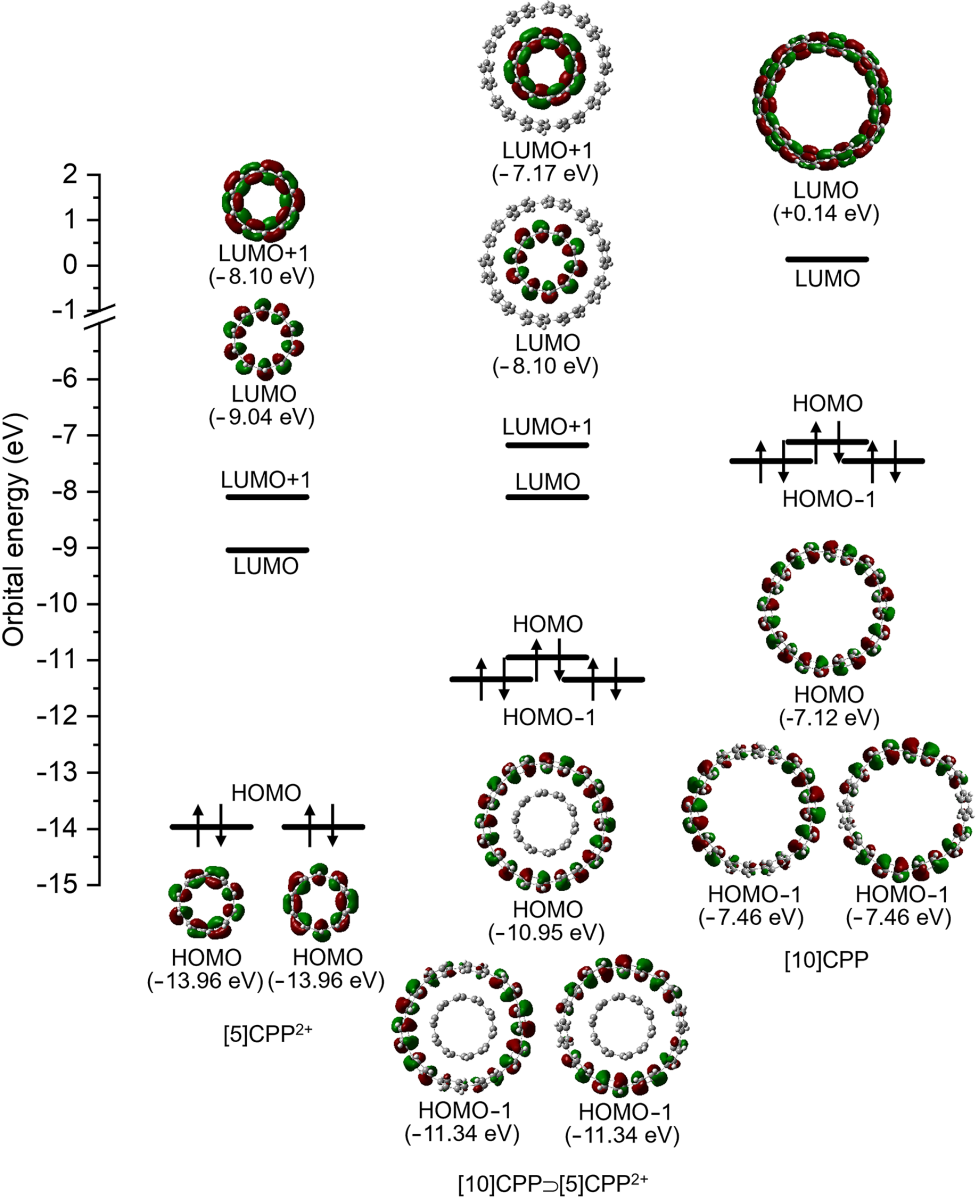
HOMO−1, HOMO, LUMO, and LUMO+1 orbitals of [10]CPP⊃[5]CPP^2+^ (**1**), [5]CPP^2+^, and [10]CPP.

In the molecular orbital analysis, the HOMO and HOMO−1s are exclusively localized on the [10]CPP host, while the LUMO and LUMO+1 are solely localized on the [5]CPP^2+^ guest (Figure 6). No orbital mixing was observed between [10]CPP and [5]CPP. However, the complex formation significantly lowered the HOMO and HOMO−1 energies derived from [10]CPP and increased the LUMO and LUMO+1 energies derived from [5]CPP^2+^. Similar changes in orbital energies are also observed for complexes **2** and **3** (see Supporting Information File 1, Figure S2), and the results are attributed to the partial electron transfer from [10]CPP to [5]CPP^2+^ in the complex. These observations also agree well with the change in oxidation and reduction potentials upon complex formation observed in the electrochemical analysis.

**Figure 7:**
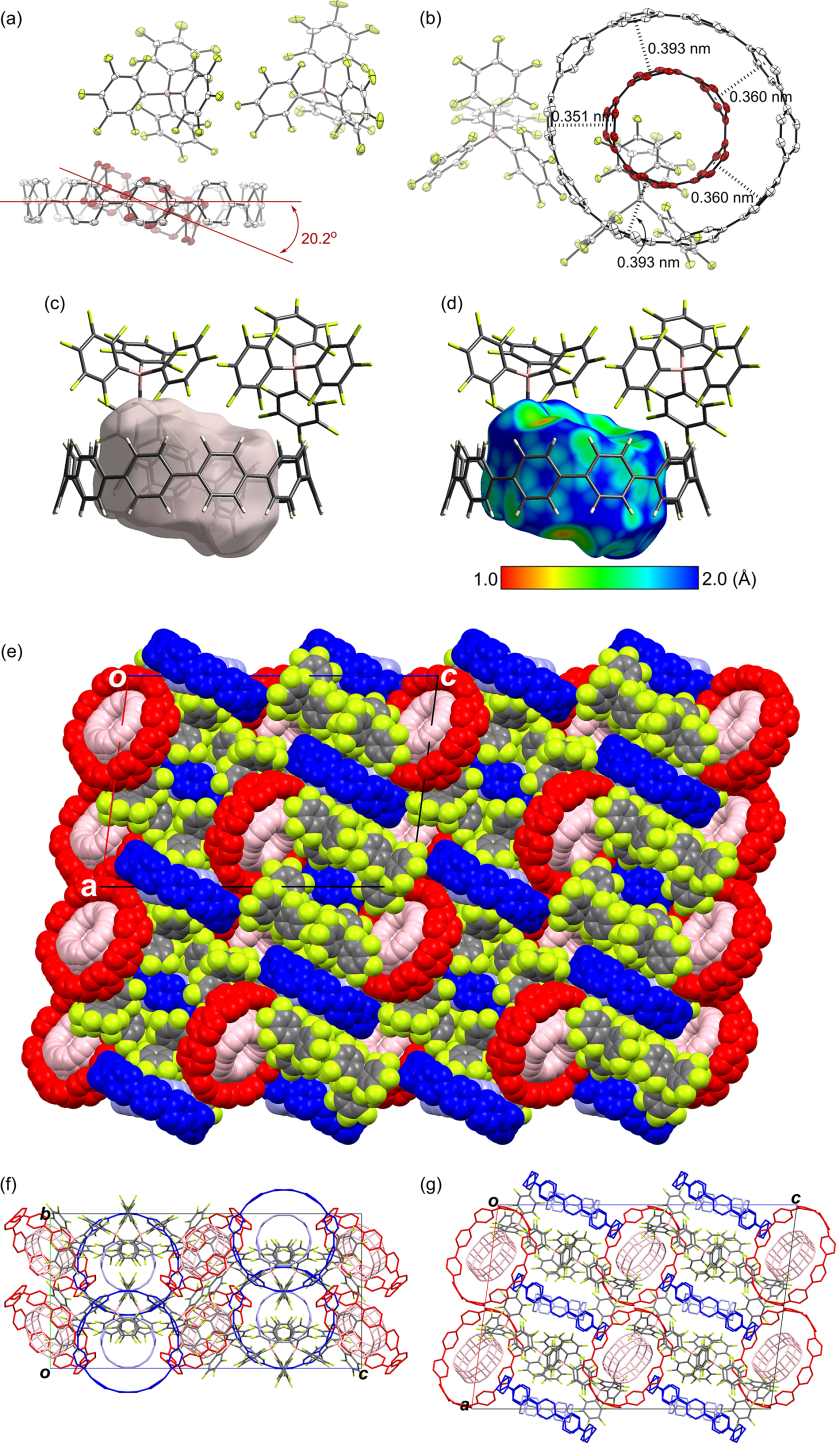
X-ray crystal structure of [10]CPP⊃[5]CPP^2+^[B(C_6_F_5_)_4_]_2_. a) Side and b) top views of ORTEP drawings. The thermal ellipsoids are scaled at the 50% probability level. All hydrogen atoms and solvent molecules are omitted for clarity. c) Hirshfeld surfaces of [5]CPP in the complex (side images). d) The Hirshfeld surfaces are colored according to the local value of *d*_e_ (distance from the surface to the nearest external atomic nuclei), and the colors range from cool (blue, 2.0 Å) to hot (red, 1.0 Å) as *d*_e_ decreases. e) Space-filling view of packing structure containing four crystalline unit lattices viewed from the *b*-axis. Wire-flame view of the packing structure of crystalline unit lattices viewed from f) the *a*-axis and g) the *b*-axis.

The double-layered structure of the complex was unambiguously determined by single-crystal X-ray analysis (Figure 7), which was performed on a crystal obtained by slow evaporation of a solution of [10]CPP⊃[5]CPP^2+^[B(C_6_F_5_)_4_^−^]_2_ in CH_2_Cl_2_ at −20 °C. In the crystal, [10]CPP encapsulated [5]CPP with an inclination angle of 20.2° (Figure 7a), and the observed structure was similar to the second most stable complex **2** obtained by the calculations. The discrepancy between the most stable calculated structure **1** and the crystalline structure is likely due to crystal packing forces, the presence of the counterion, and the inclusion of solvent molecules. The interfacial distances between the nearest neighbor centroid of a paraphenylene unit of [10]CPP and [5]CPP were in the range of 0.35–0.39 nm (Figure 7b), illustrating the importance of π–π interactions. The Hirshfeld surface of the [5]CPP guest in the complex has a cylindrical structure and is confined to the interior of [10]CPP (Figure 7c) [45–46]. In the *d*_e_ mapping (Figure 7d), which shows the distance between the Hirshfeld surface and the contacting atoms, most sp^2^-hybridzed carbon atoms of the [10]CPP host have short contact with the Hirshfeld surface, as shown in green, despite the tilting of [10]CPP and [5]CPP.

In the crystal packing, there were two orientations, as highlighted in blue and red in Figure 7e. In the packing shown in red, the host–guest complex is alternately stacked with two counterions to forms a one-dimensional (1D) columnar structure in which the complex is tilted at approximately 45° relative to the short axis (Figure 7f and Supporting Information File 1, Figure S3). Furthermore, the tilting of the complex in adjacent columnar structures with respect to the short axis is opposite to each other, as in a herringbone structure, and such columnar structures are parallel to each other through a columnar structure consisting of a host–guest complex in blue and its counterions. On the other hand, the complex in blue forms a 1D columnar structure with alternating counterions (Figure 7g), and each column is parallel to the others. Furthermore, the complex is tilted at approximately 30° relative to the long axis of the unit lattice.

## Conclusion

[10]CPP encapsulates [5]CPP^2+^ in a highly size-complementary manner, forming the corresponding host–guest complex. While several electronic structures are possible, such as [10]CPP⊃[5]CPP^2+^, [10]CPP^2+^⊃[5]CPP, and [10]CPP^•+^⊃[5]CPP^•+^, experimental and theoretical results reveal the formation of [10]CPP⊃[5]CPP^2+^ with ca. 10% CT from [10]CPP to [5]CPP^2+^. The association constant of the complex formation is about 20 times higher than that of the complex consisting of neutral [10] and [5]CPPs, and the increased association constant is ascribed to the increase of electronic interactions between the host and the guest. These findings will help tune the electronic properties of the host–guest complexes and design novel materials based on hierarchical supramolecular structures involving cyclic nanocarbons.

## Supporting Information

File 1 Experimental procedures and computation data.

File 2 Crystallographic information file for [10]CPP⊃[5]CPP^2+^[B(C_6_F_5_)_4_]_2_.

## Data Availability

All data that supports the findings of this study is available in the published article and/or the supporting information to this article. The crystal data generated and analyzed during this study is openly available in the Cambridge Crystallographic Data Centre with deposition number 2314282.
