# Supplementary material for: Enhanced host–guest interaction between [10]cycloparaphenylene ([10]CPP) and [5]CPP by cationic charges
Source: Beilstein J Org Chem. 2024 Feb 23;20:436–44. doi: 10.3762/bjoc.20.38 (PMC10896225; doi:10.3762/bjoc.20.38)
Supplement: File 1 — Experimental procedures and computation data. [file Beilstein_J_Org_Chem-20-436-s001.pdf]

BEILSTEIN JOURNAL OF ORGANIC CHEMISTRY

Supporting Information

for

Enhanced host–guest interaction between [10]cycloparaphenylene ([10]CPP) and [5]CPP by cationic charges

Eiichi Kayahara, Yoshiyuki Mizuhata and Shigeru Yamago

Beilstein J. Org. Chem. **2024,** *20,* 436–444. doi:10.3762/bjoc.20.38

Experimental procedures and computation data

License and Terms: This is a supporting information file under the terms of the Creative Commons Attribution License (https://creativecommons.org/licenses/by/4.0). Please note that the reuse, redistribution and reproduction in particular requires that the author(s) and source are credited and that individual graphics may be subject to special legal provisions.
The license is subject to the *Beilstein Journal of Organic Chemistry* terms and conditions: (https://www.beilstein-journals.org/bjoc/terms)

General. ^{1}H NMR (400 MHz) experiments were carried out in anhydrous conditions under nitrogen atmosphere for CD_2Cl_2 or 1,1,2,2-tetrachloroethane-*d*$_2$ (TCE-*d*$_2$) solution of a sample and are reported in parts per million (δ) from residual solvent peak. UV–vis–NIR spectra were measured in CH_2Cl_2 at room temperature. Cyclic voltammetry was conducted with a Pt electrode for 1,2-dichloroethane of a sample (1 mmol/L) in the presence of supporting electrolyte, $Bu_4NB(C_6F_5)_4$ (0.1 mol/L) at room temperature under nitrogen atmosphere. Scan rates of 100 mV/s were employed. The potentials were calibrated with respect to the ferrocene/ferrocenium couple (Fc/Fc^+).

Materials. [*n*]CPPs (*n* = 5, 8–12),[1] [5]CPP^{2+} $[B(C_6F_5)_4]_2$,[2] [10]CPP^{2+} $(SbF_6)_2$,[2] [5]$CPP^{\bullet+}$ $SbCl_6$,[2] and [10]$CPP^{\bullet+}$ $SbCl_6$ [2] were prepared according to literature procedures. CD_2Cl_2 or 1,1,2,2-tetrachloroethane-*d*$_2$ (TCE-*d*$_2$) was used after dried over molecular sieves.

1. Experimental procedures of titration experiment

A solution of [5]CPP^{2+} TCE-d$_2$ (1.63×10^{-2} mol L^{-1}) was added to a solution of [10]CPP^{2+} in TCE-*d*$_2$ (1.30×10^{-4} mol L^{-1}) at 50 °C. The changes in the chemical shift ($\Delta\delta_{obs}$) of [10]CPP were monitored as a function of the added ([5]CPP^{2+}), and the $\Delta\delta_{obs}$ and [[5]CPP^{2+}] were fitted by non-linear curve fitting with least squares method by using eq. 1 to obtain association constant K_a.

$$\Delta\delta_{obs} = \frac{K_a \Delta\delta_{CP}[G]}{1 + K_a[G]}$$

$\Delta\delta_{obs}$: the change in the chemical shift from free [10]CPP

$\Delta\delta_{cp}$: $\Delta\delta_{obs}$ at 100% complexation

[G]: concentration of [5]CPP^{2+}

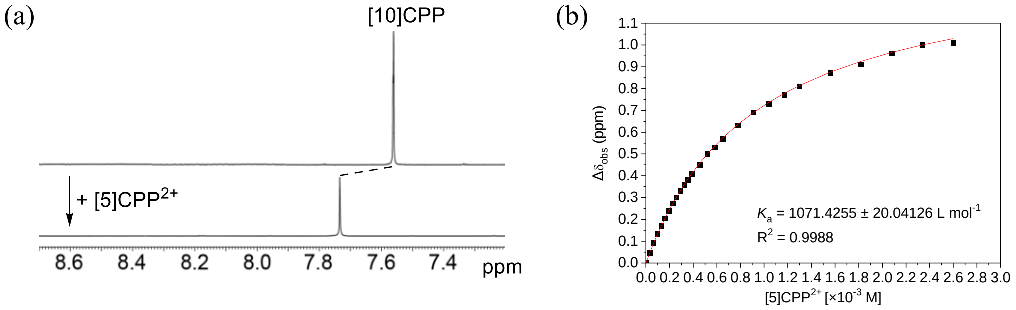

Figure S1. Titration experiments for [5]CPP. (a) ^{1}H NMR spectra of a 1:1 mixture of [5]CPP^{2+} and [10]CPP in TCE-*d*$_2$ at 25 °C, and (b) determination of K_a for [10]CPP⊃[5]CPP^{2+} by the least-square method in TCE-*d*$_2$ at 50 °C.

2. Theoretical calculations

DFT calculations were carried using Gaussian 16, Revision A.03 [3]. Geometry optimizations were performed with standard gradient techniques at ωB97X-D levels of theory with the 6-31G* basis set. All stationary points were optimized without any symmetry assumptions and characterized by normal coordinate analysis at the same level of theory.

Table S1. Energies (*E*), zero-point correction (*ZPE*), thermal correction to enthalpy (*H*) at 298.15 K, thermal correction to Gibbs free energy at 298.15 K and entropy (*S*) at 298.15 K under 1 atmosphere of pressure of the structures calculated at the (U)B3LYP/6-31G* level of theory.

Molecular	*E* (kJ mol^{-1})	*ZEP* (kJ mol^{-1})	*E* + *ZPE* (kJ mol^{-1})	*H* (kJ mol^{-1})	*G* (kJ mol^{-1})	*S* (cal mol^{-1} K^{-1})
[5]CPP^{2+}	-3030185.76	1066.709144	-3029119.05	-3029061.65	-3029245.60	147.461
[10]CPP	-6064134.93	2148.803718	-6061986.12	-6061869.27	-6062188.33	255.773
[10]CPP⊃[5]CPP^{2+} (**1**)	-9094628.84	3223.40249	-9091405.44	-9091231.27	-9091656.36	340.76
[10]CPP⊃[5]CPP^{2+} (**2**)	-9094626.31	3226.2144	-9091400.09	-9091226.76	-9091647.59	337.35
[10]CPP⊃[5]CPP^{2+} (**3**)	-9094624.61	3226.251157	-9091398.36	-9091224.48	-9091651.23	337.35

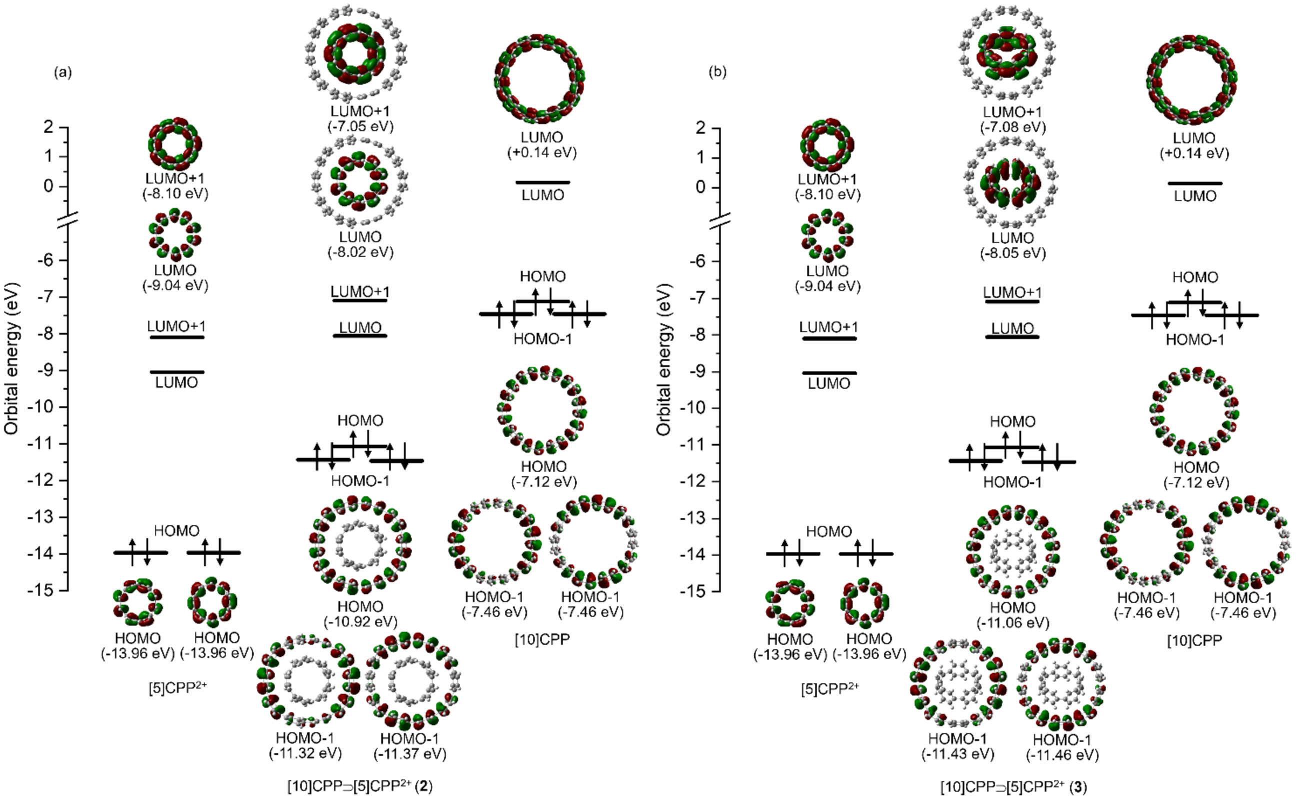

Figure S2. HOMO−1, HOMO, LUMO, and LUMO+1 orbitals of (a) [10]CPP⊃[5]CPP^{2+} (**2**), (b) [10]CPP⊃[5]CPP^{2+} (**3**), [5]CPP^{2+}, and [10]CPP.

Table S2. TD-DFT vertical one-electron excitations calculated at the ωB97X-D/6-31G* level.

Molecule	Energy (eV)	Wavelength (nm)	Oscillator Strength (f)	Description
[10]CPP⊃[5]CPP^{2+} (**1**)	0.6437	1926.10	0.0001	HOMO→LUMO (0.70157)
	1.0255	1208.99	0.0043	d-HOMO-1→LUMO (0.69947)
	1.0265	1207.88	0.0043	d-HOMO-1→LUMO (0.69104)
	1.7968	690.04	0.0547	d-HOMO-3→LUMO (0.42875)
	1.7971	689.90	0.0545	d-HOMO-3→LUMO (0.42982)
	3.0933	400.81	0.4447	d-HOMO-3→LUMO+1 (0.51169)
	3.0942	400.70	0.4443	d-HOMO-3→LUMO+1 (0.51120)
[10]CPP⊃[5]CPP^{2+} (**2**)	0.6809	1820.83	0.0001	HOMO→LUMO (0.69949)
	1.0449	1186.61	0.0010	d-HOMO-1→LUMO (0.69828)
	1.1541	1074.27	0.0043	d-HOMO-1→LUMO (0.0159)
	1.7941	691.07	0.0820	d-HOMO-3→LUMO (0.47820)
	1.8130	683.87	0.0612	d-HOMO-3→LUMO (0.53596)
	3.1295	398.74	0.2627	d-HOMO-3→LUMO+1 (0.46832)
	3.2170	396.18	0.2627	d-HOMO-3→LUMO+1 (0.46832)
[10]CPP⊃[5]CPP^{2+} (**3**)	0.8676	1428.98	0.0027	HOMO→LUMO (0.70057)
	1.1812	1049.60	0.0007	d-HOMO-1→LUMO (0.69678)
	1.7951	690.69	0.0125	HOMO→LUMO+1 (0.66245)
	1.8204	681.09	0.0869	d-HOMO-3→LUMO (0.47820)
	3.1764	390.33	0.2492	d-HOMO-3→LUMO+1 (0.43617)
	3.2853	377.39	0.2944	d-HOMO-3→LUMO+1 (0.43429)

Table S3. Averaged C–C bond lengths (Å), dihedral angles (deg), and Mullikene charge for the optimized structures of [5]CPP^{2+}, [10]CPP, and [5] and [10]CPP unit in the complex [10]CPP⊃[5]CPP^{2+}.

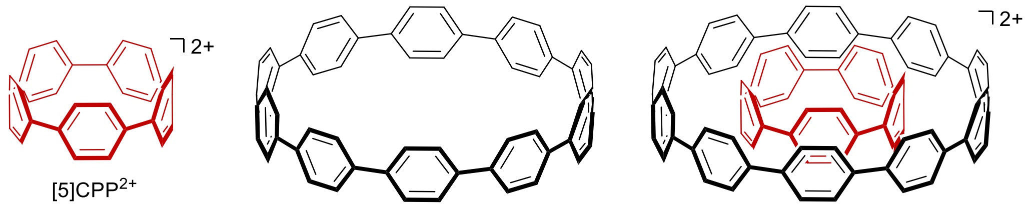

[5]CPP^{2+} [10]CPP [10]CPP⊃[5]CPP^{2+} (**1**, **2**, or **3**)

Molecule	C_{ipso}-C_{ipso} (Å)	C_{ipso}-C_{ortho} (Å)	C_{ortho}-C_{ortho} (Å)	C_{ortho}-C_{ipso}-C_{ipso}-C_{ortho} (°)	*Mulliken Charge*
[10]CPP⊃[5]CPP^{2+} (**1**)					2.00
[10]CPP	1.485	1.400	1.387	30.66	1.86
[5]CPP	1.431	1.430	1.365	0.04	0.14
[10]CPP⊃[5]CPP^{2+} (**2**)					2.00
[10]CPP	1.486	1.401	1.388	25.50	1.75
[5]CPP	1.431	1.429	1.366	0.01	0.25
[10]CPP⊃[5]CPP^{2+} (**3**)					2.00
[10]CPP	1.485	1.400	1.389	32.44	1.71
[5]CPP	1.431	1.430	1.366	1.42	0.25
Free [10]CPP	1.486	1.400	1.388	36.86	
Free [5]CPP^{2+}	1.432	1.431	1.366	0.11	
[10]CPP⊃[5]CPP^{2+} $B(C_6F_5)_4]_2$ (**X-ray**)					
[10]CPP	1.486(4)	1.399(4)	1.381(4)	17.6(4)	
[5]CPP	1.425(6)	1.429(4)	1.355(4)	1.08(4)	

3. X-ray crystallographic structures

A suitable single crystal was obtained by vapor diffusion of hexane into a sample solution in dichloroethane or dichloromethane. The intensity data were collected at 103 K on Rigaku Saturn70 CCD diffractometer with the VariMax Optix using Mo Kα radiation (λ = 0.71073 Å). The intensity data were corrected for Lorentz and polarization effects and for absorption (multiscan). The structures were solved by direct methods (SHELXT 2014/5) and refined by least-squares calculations on F^2 for all reflections (SHELXL-2019/3) [4]. The non-hydrogen atoms were refined anisotropically. Hydrogen atoms were placed using AFIX instructions. All calculations were performed by using Yadokari-XG [5] and Olex-2 1.5 [6]. The crystallographic data and the summary of solution and refinement are given in Table S4.

Table S4. Crystal data and structure refinement parameters of ([10]CPP⊃[5]CPP^{2+} [B(C_6F_5)$_4$]$_2$).

Identification code		Identification code	
Data deposition	CCDC 2314282	Density (calculated)	1.612 g/m^3
Empirical formula	$C_{60}H_{40}$, $(C_{24}BF_{20})_2$, $C_{30}H_{20}$, $(CH_2Cl_2)_{4.33}$	Absorption coefficient μ	0.326 mm^{-1}
		F(000)	11519
Formula weight	2867.21	Crystal size	0.04, 0.10, 0.15 Å
Temperature	103(2) K	θ range for data collection	1.6440 to 28.8400°
Wavelength	0.71073 Å	Index ranges	$-36<=h<=36$,
Crystal system	monoclinic		$-27<=k<=27$,
Space group	C2/c		$-55<=l<=55$
Unit cell dimensions	a = 28.4017(4) Å	Reflection collected	1777842
	b = 20.3156(3) Å	Data/restraints/parameters	28748/0/1913
	c = 41.3026(8) Å	Goodness-of-fit on F^2	1.027
	α = 90°	Final R indices [I>2σ(I)]	R_1 = 0.0575, wR_2 = 0.1120
	β = 97.433(2)°	R indices (all data)	R_1 = 0.1053, wR_2 = 0.1288
	γ = 90°		
Volume	23631.2(7) Å^3		
Z	8		

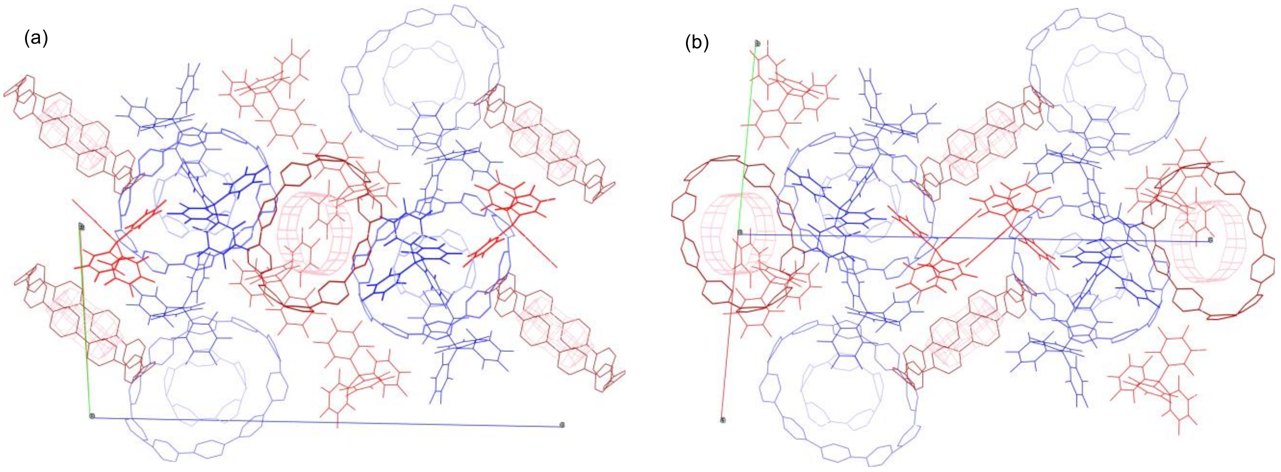

Figure S3. Wire-flame view of packing structure of crystalline unit lattices viewed from a) 45° and b)135° directions of the a, b-axes.

Cartesian Coordination

$[5]CPP^{2+}$

C	3.099	1.563	1.210
C	2.256	2.637	1.210
C	1.611	3.043	-0.001
C	2.252	2.634	-1.213
C	3.095	1.559	-1.213
C	3.339	0.839	-0.001
C	0.234	3.435	0.000
C	-0.527	3.426	1.212
C	-0.529	3.429	-1.211
C	-1.809	2.957	1.212
C	-1.811	2.959	-1.210
C	-2.396	2.472	0.001
C	-3.195	1.284	0.001
C	-3.423	0.557	1.212
C	-3.423	0.558	-1.211
C	-3.373	-0.808	1.211
C	-3.373	-0.807	-1.212
C	-3.092	-1.515	-0.001
C	-2.208	-2.642	-0.001
C	-1.587	-3.082	-1.212
C	-1.589	-3.084	1.210
C	-0.273	-3.456	-1.212
C	-0.275	-3.458	1.211
C	0.486	-3.408	0.000
C	3.392	-0.591	0.001
C	3.205	-1.331	-1.210
C	3.202	-1.328	1.213
C	2.444	-2.464	-1.210
C	2.440	-2.461	1.213
C	1.830	-2.916	0.001
H	-3.511	1.078	-2.158
H	-3.422	-1.334	2.158
H	-3.423	-1.331	-2.159
H	-2.108	-3.004	-2.160
H	-2.111	-3.009	2.157
H	3.478	1.195	2.157
H	1.989	3.093	2.157
H	1.982	3.086	-2.160
H	3.470	1.189	-2.160
H	-0.059	3.669	2.159
H	-0.062	3.674	-2.158
H	-2.323	2.841	2.160
H	-2.327	2.845	-2.157
H	-3.511	1.076	2.159
H	0.211	-3.664	-2.159
H	0.208	-3.669	2.158
H	3.556	-0.936	-2.157
H	3.548	-0.931	2.160
H	2.211	-2.938	-2.157
H	2.204	-2.932	2.160

1

C	1.960	2.836	0.674
C	1.296	3.198	1.888
C	-0.045	3.449	1.889
C	-0.797	3.353	0.676
C	-0.040	3.491	-0.529
C	1.303	3.239	-0.530
C	-2.090	2.740	0.676
C	-2.677	2.240	-0.528
C	-3.331	1.040	-0.528
C	-3.435	0.277	0.676
C	-3.293	1.021	1.889
C	-2.640	2.218	1.890
C	-3.251	-1.142	0.676
C	-2.925	-1.826	1.889
C	-1.988	-2.818	1.888
C	-1.324	-3.182	0.674
C	-2.019	-2.848	-0.530
C	-2.957	-1.854	-0.529
C	0.082	-3.446	0.673
C	0.849	-3.385	-0.532
C	2.084	-2.799	-0.533
C	2.617	-2.244	0.671
C	2.067	-2.765	1.884
C	0.835	-3.349	1.885
C	3.444	-0.243	1.884
C	3.268	1.110	1.885
C	2.943	1.796	0.673
C	3.307	1.118	-0.532
C	3.482	-0.238	-0.533
C	3.303	-0.987	0.671
H	1.819	3.154	2.836
H	-0.547	3.597	2.838
H	-0.547	3.678	-1.468
H	1.841	3.230	-1.470
H	-2.502	2.750	-1.468
H	-3.666	0.618	-1.468
H	-3.589	0.588	2.838
H	-2.436	2.701	2.838
H	-3.320	-1.483	2.838
H	-1.668	-3.234	2.836
H	-1.720	-3.295	-1.470
H	-3.386	-1.528	-1.469
H	0.406	-3.692	-1.472
H	2.601	-2.652	-1.474
H	2.562	-2.590	2.832
H	0.387	-3.621	2.834
H	3.563	-0.754	2.833
H	3.254	1.633	2.834
H	3.328	1.657	-1.472
H	3.638	-0.752	-1.474
C	-6.619	2.822	-1.437
C	-5.954	4.039	-1.437
C	-5.269	4.485	-0.297
C	-5.459	3.768	0.888
C	-6.125	2.551	0.888
C	-6.625	2.005	-0.298
C	-4.168	5.477	-0.379
C	-3.713	6.206	0.728
C	-3.380	5.509	-1.537
C	-2.457	6.801	0.728
C	-2.128	6.102	-1.537
C	-1.606	6.692	-0.379
C	-6.864	0.542	-0.380
C	-6.466	-0.138	-1.538
C	-7.230	-0.234	0.727
C	-6.288	-1.512	-1.538
C	-7.052	-1.613	0.727
C	-6.501	-2.271	-0.380
C	-5.898	-3.625	-0.297
C	-5.684	-4.414	-1.436
C	-5.275	-4.026	0.889
C	-4.731	-5.422	-1.436
C	-4.321	-5.033	0.889
C	-3.955	-5.678	-0.296
C	-0.141	6.917	-0.297
C	0.639	7.165	-1.436
C	0.535	6.610	0.888
C	2.001	6.908	-1.437

C	1.898	6.354	0.888
C	2.638	6.395	-0.297
C	3.922	5.654	-0.380
C	4.755	5.447	0.727
C	4.195	4.914	-1.537
C	5.710	4.437	0.728
C	5.148	3.907	-1.537
C	5.870	3.594	-0.379
C	-2.637	-6.356	-0.378
C	-2.011	-6.943	0.729
C	-1.868	-6.188	-1.536
C	-0.645	-7.198	0.729
C	-0.505	-6.442	-1.536
C	0.151	-6.877	-0.378
C	1.625	-6.722	-0.296
C	2.441	-6.765	-1.435
C	2.199	-6.253	0.889
C	3.695	-6.172	-1.436
C	3.453	-5.660	0.889
C	4.180	-5.513	-0.297
C	6.538	2.270	-0.296
C	7.015	1.606	-1.435
C	6.455	1.534	0.889
C	7.194	0.231	-1.435
C	6.634	0.158	0.889
C	6.902	-0.533	-0.296
C	6.594	-1.983	-0.379
C	6.653	-2.839	0.728
C	5.976	-2.471	-1.537
C	5.987	-4.059	0.728
C	5.312	-3.687	-1.537
C	5.234	-4.471	-0.379
H	-7.099	2.483	-2.350
H	-5.927	4.627	-2.350
H	-4.979	4.102	1.803
H	-6.147	1.967	1.803
H	-4.335	6.284	1.615
H	-3.709	4.980	-2.427
H	-2.124	7.332	1.616
H	-1.511	6.022	-2.427
H	-6.200	0.424	-2.429
H	-7.631	0.246	1.615
H	-5.889	-1.988	-2.429
H	-7.317	-2.180	1.615
H	-6.235	-4.209	-2.349
H	-5.445	-3.466	1.804
H	-4.555	-5.984	-2.349
H	-3.771	-5.232	1.804
H	0.167	7.516	-2.349
H	-0.028	6.448	1.804
H	2.568	7.065	-2.350
H	2.364	5.999	1.803
H	4.636	6.062	1.615
H	3.591	5.063	-2.428
H	6.317	4.285	1.616
H	5.263	3.296	-2.427
H	-2.592	-7.175	1.618
H	-2.321	-5.762	-2.428
H	-0.187	-7.625	1.618
H	0.071	-6.209	-2.427
H	2.074	-7.225	-2.348
H	1.614	-6.239	1.804
H	4.283	-6.180	-2.349
H	3.814	-5.199	1.803
H	7.204	2.163	-2.348
H	6.128	2.019	1.805
C	-6.814	-1.422	-0.222
C	-2.896	-6.622	-1.175
H	7.518	-0.260	-2.348
H	6.441	-0.394	1.804
H	7.201	-2.537	1.616
H	5.932	-1.851	-2.427
H	6.028	-4.684	1.616
H	4.766	-3.985	-2.428

2

C	-1.904	-3.121	-0.552
C	-3.160	0.678	2.086
C	-3.064	1.395	0.851
C	-1.295	-2.863	1.775
C	-3.483	-0.688	-0.311
C	-2.200	-2.542	0.719
C	0.401	-3.415	0.134
C	-0.026	-3.284	1.489
C	-0.642	-3.558	-0.834
C	-3.485	0.682	-0.310
C	-1.911	3.118	-0.550
C	-2.206	2.537	0.721
C	-1.301	2.860	1.777
C	-3.158	-0.686	2.085
C	-3.061	-1.402	0.850
C	3.430	-1.445	0.504
C	1.957	2.557	-1.578
C	1.705	2.985	-0.237
C	2.723	-1.443	-1.812
C	3.426	1.451	0.505
C	3.281	-0.710	-0.716
C	1.712	-2.982	-0.238
C	1.963	-2.553	-1.579
C	2.666	-2.551	0.737
C	2.660	2.556	0.738
C	-0.033	3.284	1.491
C	0.394	3.415	0.136
C	-0.650	3.557	-0.832
C	2.720	1.449	-1.811
C	3.279	0.717	-0.715
C	-5.729	3.281	-1.449
C	-6.376	2.824	-0.294
C	-2.807	-5.935	1.111
C	-3.521	-5.991	-0.090
C	-4.934	4.418	-1.421
C	-7.213	0.687	-1.358
C	-1.560	-6.996	-1.111
C	-6.596	0.687	0.956
C	-1.474	-6.309	1.174
C	-5.535	-4.801	0.863
C	-5.723	-3.292	-1.448
C	-6.817	1.409	-0.223
C	-0.789	-6.754	0.037
C	-6.594	-0.699	0.956
C	-5.546	4.791	0.861
C	-4.733	-5.146	-0.233
C	-6.339	-3.669	0.833
C	1.388	-6.562	-1.203
C	-4.925	-4.428	-1.419
C	-7.211	-0.701	-1.358
C	-6.348	3.658	0.831
C	1.457	-6.572	1.179
C	-6.370	-2.836	-0.293
C	0.699	-6.710	0.008
C	-3.533	5.985	-0.091
C	-4.744	5.137	-0.234
C	-2.909	6.616	-1.176
C	-2.819	5.930	1.110
H	-7.487	-1.230	-2.265
H	-6.923	3.394	1.714

C	2.758	6.129	1.142
C	3.399	5.833	-0.068
C	5.800	-3.141	-1.162
C	6.434	-2.789	0.036
C	1.443	6.574	1.179
C	5.408	4.758	1.050
C	5.417	-4.747	1.050
C	4.919	4.224	-1.225
C	4.928	-4.214	-1.226
C	7.432	-0.686	-0.934
C	6.707	0.701	1.346
C	4.631	4.997	-0.095
C	4.641	-4.988	-0.096
C	5.793	3.152	-1.161
C	1.375	6.565	-1.203
C	6.959	-1.407	0.170
C	7.431	0.701	-0.934
C	2.770	-6.124	1.142
C	6.709	-0.688	1.346
C	6.284	3.682	1.117
C	2.701	6.156	-1.240
C	2.713	-6.150	-1.240
C	6.956	1.421	0.170
C	3.411	-5.826	-0.068
C	-0.803	6.752	0.037
C	0.685	6.711	0.008
C	-1.488	6.306	1.173
C	-1.574	6.993	-1.111
C	6.428	2.802	0.036
C	6.291	-3.669	1.116
H	-2.663	-3.137	-1.326
H	-3.122	1.207	3.032
H	-1.543	-2.628	2.803
H	-3.655	-1.216	-1.241
H	0.691	-3.373	2.297
H	-0.416	-3.919	-1.830
H	-3.658	1.210	-1.240
H	-2.671	3.133	-1.323
H	-1.548	2.623	2.805
H	-3.120	-1.215	3.031
H	4.070	-1.063	1.291
H	1.428	3.015	-2.405
H	2.784	-1.054	-2.822
H	4.067	1.071	1.291
H	1.435	-3.012	-2.407
H	2.716	-3.038	1.703
H	2.710	3.042	1.705
H	0.684	3.374	2.299
H	-0.425	3.919	-1.828
H	2.781	1.061	-2.821
H	-5.764	2.688	-2.359
H	-3.261	-5.483	1.988
H	-4.368	4.684	-2.310
H	-7.490	1.214	-2.266
H	-1.108	-7.449	-1.988
H	-6.308	1.210	1.863
H	-0.944	-6.141	2.104
H	-5.497	-5.397	1.771
H	-5.759	-2.700	-2.358
H	-6.305	-1.221	1.863
H	-5.509	5.387	1.768
H	-6.914	-3.406	1.716
H	0.873	-6.710	-2.147
H	-4.360	-4.693	-2.308
H	-1.024	-4.086	0.957
H	-3.001	-3.087	0.041
H	-3.026	-0.186	3.244
H	-1.050	-1.205	4.177
H	1.010	-6.771	2.147
H	-3.458	6.776	-2.100
H	-3.273	5.477	1.987
H	-3.445	-6.783	-2.099
H	3.265	5.956	2.086
H	5.901	-2.500	-2.033
H	0.996	6.771	2.148
H	5.306	5.395	1.923
H	5.317	-5.385	1.922
H	4.376	4.381	-2.151
H	4.386	-4.373	-2.151
H	7.750	-1.215	-1.829
H	6.399	1.227	2.246
H	5.895	2.512	-2.032
H	0.860	6.712	-2.146
H	7.747	1.230	-1.828
H	3.278	-5.951	2.086
H	6.401	-1.215	2.246
H	6.832	3.504	2.038
H	3.177	6.046	-2.209
H	3.188	-6.038	-2.209
H	-0.957	6.139	2.104
H	-1.123	7.446	-1.988
H	6.839	-3.490	2.037

3

C	2.579	1.550	-1.312
C	3.099	0.300	-1.790
C	3.556	-0.631	-0.906
C	3.496	-0.359	0.499
C	3.497	1.022	0.865
C	3.040	1.956	-0.020
C	3.025	-1.359	1.411
C	2.574	-0.997	2.716
C	1.455	-1.581	3.244
C	0.729	-2.549	2.488
C	1.450	-3.197	1.444
C	2.573	-2.618	0.920
C	-0.712	-2.553	2.487
C	-1.429	-3.206	1.445
C	-2.556	-2.634	0.920
C	-3.015	-1.378	1.410
C	-2.565	-1.011	2.714
C	-1.443	-1.589	3.242
C	-3.493	-0.381	0.498
C	-3.504	1.000	0.863
C	-3.054	1.937	-0.022
C	-2.589	1.533	-1.313
C	-3.100	0.280	-1.791
C	-3.551	-0.654	-0.907
C	-0.685	1.439	-2.902
C	0.677	1.444	-2.901
C	1.398	2.122	-1.863
C	0.672	3.150	-1.173
C	-0.693	3.146	-1.174
C	-1.411	2.113	-1.864
H	3.026	0.038	-2.840
H	3.851	-1.614	-1.256
H	3.716	1.327	1.881
H	2.909	2.979	0.318
H	3.030	-0.169	3.246
H	1.061	-1.200	4.179
H	1.050	-4.079	0.956
H	3.021	-3.067	0.040
C	-2.847	6.302	0.251
C	-0.720	6.568	-1.004
C	-1.440	6.771	0.179
C	-3.705	6.313	-0.857

H	-3.726	1.304	1.879
H	-2.930	2.960	0.316
H	-3.025	0.017	-2.841
H	-3.839	-1.640	-1.257
H	-1.206	0.816	-3.620
H	1.202	0.824	-3.619
H	1.192	3.879	-0.560
H	-1.218	3.872	-0.561
C	3.722	-6.204	0.610
C	4.856	-5.399	0.640
C	5.153	-4.529	-0.418
C	4.356	-4.617	-1.567
C	3.221	-5.415	-1.593
C	2.839	-6.170	-0.477
C	6.046	-3.351	-0.270
C	6.644	-2.723	-1.373
C	6.073	-2.668	0.950
C	7.066	-1.402	-1.301
C	6.494	-1.346	1.021
C	6.904	-0.655	-0.125
C	1.441	-6.671	-0.410
C	0.720	-6.573	0.787
C	0.720	-6.998	-1.567
C	-0.671	-6.578	0.786
C	-0.667	-7.003	-1.567
C	-1.391	-6.681	-0.411
C	-2.792	-6.190	-0.478
C	-3.676	-6.231	0.609
C	-3.179	-5.438	-1.594
C	-4.815	-5.434	0.639
C	-4.320	-4.648	-1.568
C	-5.118	-4.566	-0.419
C	6.877	0.830	-0.128
C	7.079	1.592	1.032
C	6.389	1.494	-1.258
C	6.653	2.913	1.105
C	5.959	2.811	-1.184
C	6.003	3.520	0.022
C	5.124	4.706	0.179
C	4.796	5.545	-0.894
C	4.366	4.830	1.350
C	3.656	6.338	-0.859
C	3.222	5.616	1.383
C	2.799	6.321	0.249
C	-6.020	-3.394	-0.271
C	-6.623	-2.770	-1.374
C	-6.052	-2.711	0.949
C	-7.054	-1.453	-1.302
C	-6.482	-1.393	1.020
C	-6.898	-0.705	-0.125
C	-6.883	0.780	-0.128
C	-7.091	1.541	1.032
C	-6.399	1.448	-1.257
C	-6.675	2.864	1.106
C	-5.980	2.769	-1.183
C	-6.030	3.476	0.023
C	1.388	6.780	0.178
C	0.667	7.170	1.315
C	0.669	6.573	-1.004
C	-0.721	7.165	1.316
C	-3.265	5.593	1.384
C	-4.839	5.511	-0.892
C	-4.403	4.798	1.351
C	-5.160	4.669	0.180
H	3.505	-6.839	1.464
H	5.495	-5.419	1.518
H	4.566	-3.979	-2.421
H	2.573	-5.372	-2.464
H	6.740	-3.258	-2.314
H	5.648	-3.130	1.837
H	7.484	-0.932	-2.186
H	6.387	-0.814	1.962
H	1.244	-6.388	1.721
H	1.248	-7.200	-2.494
H	-1.197	-6.396	1.720
H	-1.194	-7.209	-2.494
H	-3.454	-6.865	1.463
H	-2.532	-5.390	-2.464
H	-5.455	-5.459	1.516
H	-4.535	-4.011	-2.421
H	7.547	1.140	1.902
H	6.236	0.947	-2.183
H	6.789	3.463	2.031
H	5.480	3.255	-2.052
H	5.413	5.544	-1.788
H	4.607	4.217	2.215
H	3.407	6.946	-1.724
H	2.599	5.601	2.272
H	-6.714	-3.306	-2.315
H	-5.623	-3.170	1.836
H	-7.476	-0.986	-2.187
H	-6.380	-0.861	1.962
H	-7.556	1.084	1.902
H	-6.243	0.903	-2.183
H	-6.816	3.413	2.032
H	-5.505	3.217	-2.051
H	1.197	7.435	2.226
H	1.193	6.300	-1.916
H	-1.252	7.426	2.227
H	-1.243	6.292	-1.915
H	-3.461	6.924	-1.722
H	-2.641	5.582	2.274
H	-5.456	5.506	-1.786
H	-4.640	4.184	2.216

References

1. Patel, V. K.; Kayahara, E.; Yamago, S. *Chem. Eur. J.* **2015**, *21* (15), 5742-5749.
2. Kayahara, E.; Kouyama, T.; Kato, T.; Yamago, S. *J. Am. Chem. Soc.* **2016**, *138* (1), 338-344.
3. Frisch, M. J.; Trucks, G. W.; Schlegel, H. B.; Scuseria, G. E.; Robb, M. A.; Cheeseman, J. R.; Scalmani, G.; Barone, V.; Petersson, G. A.; Nakatsuji, H.; Li, X.; Caricato, M.; Marenich, H. P.; Bloino, J.; Janesko, B. G.; Comperts, R.; Mennucci, B.; Hratchian, H. P.; Ortiz, J. V.; Izmaylov, A. F.; Sonnenberg, J. L.; Williams-Young, D.; Ding, F.; Lipparini, F.; Egidi, F.; Goings, J.; Peng, B.; Petrone, A.; Henderson, T.; Ranasinghe, D.; Zakrzewski, V. G.; Gao, J.; Rega, N.; Zheng, G.; Liang, W.; Hada, M.; Ehara, M.; Toyota, K.; Fukuda, R.; Hasegawa, J.; Ishida, M.; Nakajima, T.; Honda, Y.; Kitao, O.; Nakai, H.; Vreven, T.; Throssell, K.; Montgomery, J., J. A.; Peralta, J. E.; Ogliaro, F.; Bearpark, M. J.; Heyd, J. J.; Brothers, E. N.; Kudin, K. N.; Staroverov, V. N.; Keith, T. A.; Kobayashi, R.; Normand, J.; Raghavachari, K.; Rendell, A. P.; Burant, J. C.; Iyengar, S. S.; Tomasi, J.; Cossi, M.; Millam, J. M.; Klene, M.; Adamo, C.; Cammi, R.; Ochterski, J. W.; Martin, R. L.; Morokuma, K.; Farkas, O.; Foresman, J. B.; Fox, D. J. *Gaussian 16, Revision A.03*; Gaussian Inc: Wallingford CT, 2016.
4. Sheldrick, G. M. *Acta Cryst.* **2015**, *A71*, 3-8.
5. Kabuto, C.; Akine, S.; Kwon, E. *J. Cryst. Soc. Jpn.* **2009**, *51* (3), 218-224.
6. Dolomanov, O. V.; Bourhis, L. J.; Gildea, R. J.; Howard, J. A. K.; Puschmann, H. *J. Appl. Cryst.* **2009**, *42* (2), 339-341.
